# Rapid recurrence of spindle cell type undifferentiated carcinoma early after radical surgery in a bile duct cancer patient – A case report

**DOI:** 10.1016/j.ijscr.2021.105800

**Published:** 2021-03-19

**Authors:** Hiroki Kajioka, Atsushi Muraoka

**Affiliations:** Department of Surgery, Kagawa Rosai Hospital, 3-3-1 Joto-cho, Marugame-shi, Kagawa, 763-8502, Japan

**Keywords:** Undifferentiated carcinoma, Cholangiocarcinoma, Spindle cell, Bile outflow, Chemotherapy, Case report

## Abstract

**Introduction:**

Spindle cell type undifferentiated carcinoma of the extrahepatic bile duct is extremely rare and has a poor prognosis. However, its pathology is not fully known, yet.

**Case presentation:**

76-year-old man with abdominal pain and dark-colored urine was referred to our department. Contrast-enhanced computed tomography showed an enhanced tumor at the junction of the cystic duct and direct invasion of the portal vein. He was diagnosed as having resectable biliary cancer and underwent a subtotal stomach-preserving pancreaticoduodenectomy with a reconstruction of the portal vein. Histopathological findings demonstrated undifferentiated spindle cell carcinoma. Forty-two days post-surgery, he presented with peritoneal dissemination and local recurrence with ascites, and died sixty-five days after his operation.

**Clinical discussion:**

Spindle cell type undifferentiated carcinoma has highly metastatic potentials and also easily invade adjacent organs. Therefore, the prognosis of an undifferentiated, spindle cell type cholangiocarcinoma was poor. Although only surgery ensures cure, multidisciplinary treatment, including chemotherapy and radiotherapy is required.

**Conclusion:**

Although surgery for spindle cell type undifferentiated carcinoma may provide a cure, we must consider the induction of multidisciplinary treatment.

## Highlights

• An undifferentiated, spindle cell type cholangiocarcinoma (USCC) is extremely rare.
• USCC possesses a high metastatic potential.
• Upfront surgery for USCC was not feasible.
• USCC may be the induction of multidisciplinary treatment.

## 1 Introduction

Cholangiocarcinoma can be pathologically divided into three types: tubular, mucinous, and adenosquamous adenocarcinoma. Furthermore, its variants include clear cell, pleomorphic, and spindle cell types [1]. Among these, the undifferentiated spindle cell carcinoma, arising from the extrahepatic bile duct, is extremely rare [2]. To the best of our knowledge, only eleven cases of spindle cell type undifferentiated cholangiocarcinoma have been reported previously [[3], [4], [5], [6], [7], [8], [9], [10], [11], [12], [13]]. The pathology of this cancer is not fully known, yet. Here, we report a case of spindle cell type undifferentiated cholangiocarcinoma with recurrence at the cholejejunostomy region and rapid peritoneal dissemination after surgery. The work has been presented in line with the SCARE guideline [14].

## 2 Presentation of case

**Fig. 1:**
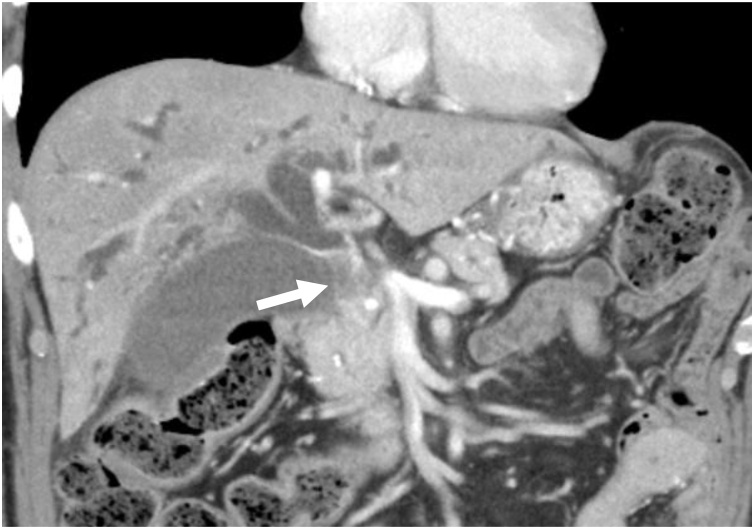
CECT showing an enhanced tumor measuring 33 × 13 mm, with intrahepatic duct dilatation, at the junction of the cystic duct (a: arrow).

A 76-year-old Japanese man had experienced epigastric pain and consulted his previous doctor 1 month before referral to our hospital. He had been diagnosed with gastritis and administered proton-pump inhibitor. After half a month, he had presented with dark-colored urine and jaundice. Abdominal ultrasound and CT had shown stricture of the middle bile duct with intrahepatic duct dilation. Therefore, the previous doctor had performed an endoscopic retrograde cholangiography and placed a double-pigtail endoscopic retrograde biliary drainage. Subsequently, he had been referred to our hospital for further examination. Physical examination revealed icteric sclerae and epigastric tenderness. The patient had a history of diabetes mellitus for more than ten years, along with insulin induction. However, he had no allergies, no relevant family history and was not a habitual drinker or smoker. Liver function tests showed high levels of serum aspartate aminotransferase (60 IU/L), serum alanine aminotransferase (89 IU/L), serum alkaline phosphatase (1035 IU/L), serum gamma-glutamyl transpeptidase (161 IU/L), serum total bilirubin (25.87 mg/dL), and serum direct bilirubin (21.12 mg/dL). The serum level of carcinoembryonic antigen was within the normal range, but carbohydrate antigen 19−9 was increased (136 IU/mL). Cholangiography showed complete obliteration of the middle bile duct and a dilated intrahepatic duct. Contrast-enhanced computed tomography (CECT) showed an enhanced tumor measuring 33 × 13 mm at the junction of the cystic duct (Fig. 1) during the arterial and portal phase with regional lymph node swelling. Magnetic resonance cholangiopancreatography showed a tumor with high intensity on T2-weighted imaging, low intensity on T1-weighted imaging, and prominently high intensity on diffusion-weighted imaging. Additionally, bile juice cytology showed poorly differentiated adenocarcinoma. The patient was then diagnosed as having cholangiocarcinoma, and he underwent a subtotal stomach-preserving pancreaticoduodenectomy (SSPPD) with the reconstruction of the portal vein.

**Fig. 2:**
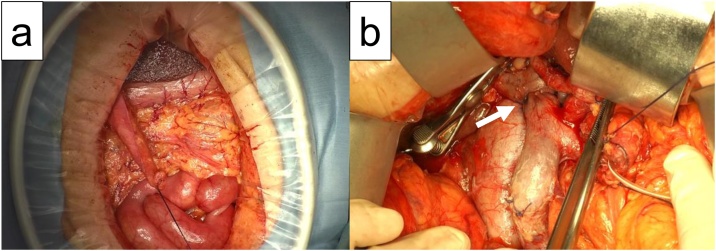
Intraoperative findings showing absence of macroscopical dissemination (a). Findings after tumor resection and the reconstruction of portal vein (arrow) (b).

Intraoperative findings revealed that the nodule was in the middle of the bile duct, penetrating both the cystic duct and portal vein. No peritoneal dissemination was found macroscopically (Fig. 2a), and no malignant cells were found on the lavage cytology. However, a swollen lymph node (LN) at LN station 12b was noted. We proceeded with the planned SSPPD and end-to-end anastomosis of the portal vein with 5–0 Prolene running suture, performed by a hepato-biliary-pancreatic surgeon (Fig. 2b). During the cholejejunostomy, some bile juice flowed out from the hepatic duct. However, before laparotomy closure, abdominal cavity was lavaged using normal saline 5000 ml.

**Fig. 3:**
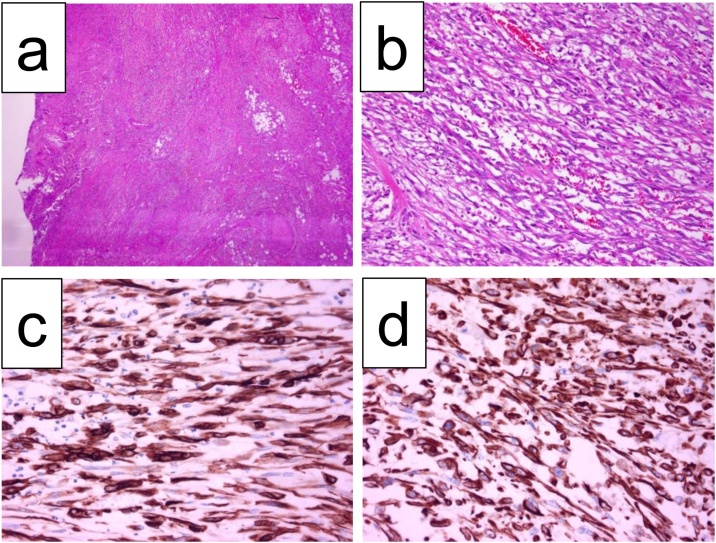
Histopathological findings showing poorly differentiated carcinoma composed of spindle and polygonal cells (**a:** ×12.5, **b**: ×200), positive for cytokeratin AE1/AE3 (**c**) and vimentin (**d**). HE staining.

The resected specimen showed the nodule, which was 33 × 20 mm in size, at the junction of the cystic duct. Moreover, the nodule had directly invaded into the duodenum and pancreas. Histopathological examination showed an undifferentiated carcinoma composed of spindle and polygonal cells (Fig. 3a, b), without involvement of the portal vein and with nerve invasion and an LN metastasis at LN station 12b, which was the nearest to the primary lesion, stage IIb (T2N1M0). Both the stump of the bile duct and surface of the resected specimen was negative for malignant cells. Immunohistochemical studies showed that the tumor cells were positive for both cytokeratin AE1/AE3 and vimentin (Fig. 3c, d). Based on these findings, the tumor was diagnosed as spindle cell type undifferentiated carcinoma with complete resection.

**Fig. 4:**
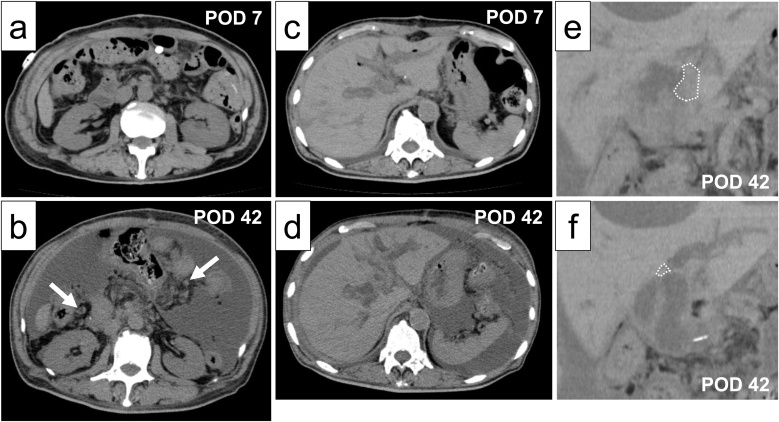
Abdominal CT scan showing multiple peritoneal nodules (arrow) with a large amount of ascites at POD42 (b) compared to POD 7 (a) and dilation of the intrahepatic bile duct at POD 42 (d) compared to POD 7(c). Local recurrence at the site of the cholejejunostomy is shown (e, f). POD: postoperative day.

Post-surgery, his pain was controlled with epidural anesthesia. He was discharged from the intensive care unit, and mobilization was initiated 1 day post-surgery. Antibiotics were administered until the day after the surgery. In the absence of findings of pancreatic fistula, drainage tube was removed 3 days post-surgery. The next day, oral food intake was initiated and non-steroidal anti-inflammatory drug were administered instead of epidural anesthesia. He was discharged from the hospital seventeen days post operation without any complications (Table 1). However, forty-two days post-operation, the patient was readmitted to the hospital and presented with jaundice, abdominal distension, and fever; the patient did not have heart failure. Liver function tests demonstrated high levels of serum aspartate aminotransferase (771 IU/L), serum alanine aminotransferase (135 IU/L), serum alkaline phosphatase (5912 IU/L), serum gamma-glutamyl transpeptidase (584 IU/L), and serum total bilirubin (8 mg/dL) and low levels of serum total protein (6.1 mg/dL) and albumin (2.5 mg/dL) at readmission. CT findings showed massive ascites with some peritoneal nodules forty-two days post-operation compared with seven days post-operation (Fig. 4a, b) and an iso-density area at the site of the cholejejunostomy (Fig. 4e, f), accompanied by intrahepatic dilatation (Fig. 4c, d). Additionally, ascites cytology was positive for malignant cells, and biochemistry showed low levels of total protein (3.5 mg/dL) and albumin (1.7 mg/dL). Peritoneal dissemination and local recurrence were clinically diagnosed. Because of his severely deteriorating liver function, chemotherapy was not administered. The patient died 65 days after his operation.

**Table 1:** Laboratory parameters.

Parameters		Before surgery	POD1	POD5	POD7	POD16
Temperature	°F	96.6	97.2	98.1	97.5	97
WBC	(/ul)	4900	11900	7600	7500	5900
RBC	(×10^4^/ul)	436	394	356	406	399
Hemoglobin	(g/dL)	13.7	12.7	11.5	12.9	12.4
Hematocrit	(%)	40.2	37.3	32.6	36.9	36.6
AST	(IU/L)	21	1246	27	15	17
ALT	(IU/L)	19	895	129	71	14
ALP	(IU/L)	331	400	246	265	277
Total bilirubin	(mg/dL)	2.6	1.8	1.6	1.3	0.9
Direct bilirubin	(mg/dL)	1.8	1	0.8	0.7	0.5
γ-GTP	(IU/L)	27	211	90	83	51
BUN	(mg/dL)	11	26	15	10	15
Creatinine	(mg/dL)	0.72	1.75	1.13	1.12	1.08
Na	(mEq/L)	140	142	132	133	138
K	(mEq/L)	4.4	4.2	4.4	4.6	4
Cl	(mEq/L)	101	110	99	98	102
Ca	(mg/dL)	8.8	9.1	7.3	7.7	8.5
CRP	(mg/dL)	0.1	7.8	9.6	5.4	0.3

POD: postoperative day, WBC: white blood cell, RBC: red blood cell.

AST: asparate aminotransferase, ALT: alanine aminotransferase, BUN: blood urea nitrogen.

## 3 Discussion

Cholangiocarcinoma is pathologically divided into subgroups according to cell differentiation (well-differentiated, moderately differentiated, and poorly differentiated). Additionally, poorly differentiated carcinomas are further subdivided into clear cell, pleomorphic, and spindle cell types [1]. Albores-Saavedra et al. reported that the spindle cell type makes up only 0.38% of carcinoma cases [2]. A literature search in PubMed showed that only 12 cases of spindle cell type undifferentiated cholangiocarcinoma, including the present case, have been reported [[3], [4], [5], [6], [7], [8], [9], [10], [11], [12], [13]] (Table 2). Spindle cell type undifferentiated cholangiocarcinoma is reported to be associated with poor prognosis [3,6,7,10,12,13]. Spindle cell type undifferentiated cholangiocarcinoma has high metastatic potential, resulting in poor prognosis. Saentaweesuk et al. reported that, *in vitro*, the upregulation of vimentin expression in a cholangiocarcinoma cell line increased migration and invasion abilities. This upregulated cell line showed aggressive features of cancer and promoted lung metastasis *in vivo* [14]. Our patient’s prognosis was consistent with this high metastatic potency; the present patient died due to peritoneal dissemination and local recurrence 65 days post-operation. Among the already reported cases, postoperative outcomes of tumors located above the middle bile duct, excluding cases in which patients died due to other diseases, were worse (Table 1). Additionally, two cases involving the middle bile duct, in which patients died, including the present case, had lymph node metastasis and nerve invasion. These findings demonstrated that the tumor location of undifferentiated, spindle cell type cholangiocarcinoma may influence tumor staging and be associated with patient’s outcome.

**Table 2:** Previously reported cases of spindle cell type undifferentiated cholangiocarcinoma.

Case No.	Year	Age/Sex	Tumor Size (cm)	Tumor Location	Surgical procedure	Postoperative Outcome
1^3)^	1994	52/F	4.0	distal	PD	15 months, alive
2^4)^	1995	62/M	3.5	hepatic hilus	ELHL	10 days, dead
3^5)^	2000	81/M	9.2	middle	PPPD	10 months, dead
4^6)^	2002	78/M	1.0	distal	PD	15 months, alive
5^7)^	2003	71/M	1.2	distal	PD	60 months, alive
6^8)^	2004	78/M	4.0	distal	PD	ND
7^9)^	2007	59/M	4.0	hepatic hilus	RTH	11 days, dead
8^10)^	2007	61/M	4.8	middle	PPPD	7 months, alive
9^11)^	2010	65/M	1.5	middle	PD	ND
10^12)^	2012	67/M	1.7	proximal	BDR	16 months, alive
11^13)^	2017	51/F	3.8	distal	PD	3 years, alive
12	Present case	76/M	3.3	middle	SSPPD	65 days, dead

Preoperative precise diagnosis with undifferentiated, spindle cell type cholangiocarcinoma could be difficult. Among the reported cases, accurate diagnosis could not be made in all cases preoperatively. Radiological findings of this kind of tumor are not specific. Therefore, repeated biopsies and bile juice cytology are indispensable to accurate diagnosis. In the present case, we could have performed accurate preoperative diagnosis if repeated biopsies and bile juice cytology for cholangiocarcinoma were attempted.

Although we performed the surgery with an aim to cure, the present case showed an early relapse. Considering this case, there were two possible causes of cancer relapse; preoperative or intraoperative dissemination. In the present case, the tumor involved the surrounding fat tissue and showed LN metastasis. These findings may have indicated cancer spread preoperatively and subclinically. In addition, surgical inflammation stimulated cancer cells, followed by early cancer early recurrence. Nevertheless, the cut surface and bile duct stump of the resected specimen were negative for malignant cells. In addition, intraoperative findings indicated no peritoneal dissemination and no other bile duct cancers. Moreover, during the cholejejunostomy, some bile outflow occurred. These findings indicate that cancer cells in the bile juice may have promoted peritoneal dissemination with a local recurrence at the cholejejunostomy site. In any mechanism, in this case, the operation was technically successful, but from an oncological perspective, the operation did not appear feasible.

According to the Consensus statement of the European network, chemotherapy is recommended for patients with unresectable cholangiocarcinoma, or for local recurrence after resection of cholangiocarcinoma, or as adjuvant chemotherapy [15]. However, the analysis of surveillance, epidemiology and end results program database showed that cholangiocarcinoma patients with positive regional LNs had similar overall survival after resection when compared with that after adjuvant systemic chemotherapy [16]. On the contrary, in a national database study, a propensity score matched analysis demonstrated that the 5-year OS rates for the neoadjuvant chemotherapy group were 42.5% and 31.7% for the adjuvant chemotherapy group [17]. Neoadjuvant chemotherapy may attenuate cholangiocarcinoma.

Spindle cell type undifferentiated cholangiocarcinoma overexpress vimentin which has increased migration and invasion abilities [18], leading to metastasis. Spindle cell type undifferentiated cholangiocarcinoma has metastatic potentials compared with differentiated cholangiocarcinoma. Only the operation is not enough to ensure cure. Therefore, we should consider multidisciplinary treatment, such as neoadjuvant chemotherapy. Although the effectiveness of neoadjuvant chemotherapy for undifferentiated carcinoma remains unclear due to varied responses in reported literature, Jones et al. reported that neoadjuvant chemotherapy decreased tumor size and conferred possible survival benefits for patients [19]. Therefore, neoadjuvant chemotherapy may improve outcomes in patients with undifferentiated, spindle cell type cholangiocarcinoma.

## 4 Conclusion

We report a case of the rapid recurrence of spindle cell type undifferentiated cholangiocarcinoma. The prognosis was found to be poor, especially with its location above the middle bile duct, due to its metastatic potential. It was difficult to improve overall survival with upfront surgery for spindle cell type undifferentiated cholangiocarcinoma. Therefore, we must consider the induction of multidisciplinary treatment as spindle cell type undifferentiated cancer possesses a high metastatic potential.

## Declaration of Competing Interest

The authors report no declarations of interest.

## Sources of funding

The author listed have no source of funding to disclose.

## Ethical approval

Our institutional review board does not require case reports to be submitted for ethical approval.

## Consent

Written informed consent was obtained from the legally authorized representative of the deceased patient to publish the patient’s anonymized information. A copy of the written consent is available for review by the Editor-in-Chief of this journal on request.

## Author contribution

Hiroki Kajioka: performing the operation, writing the paper.

Atsushi Muraoka: reviewing the paper.

## Registration of research studies

Not applicable.

## Guarantor

Hiroki Kajioka.

## Provenance and peer review

Not commissioned, externally peer-review.
